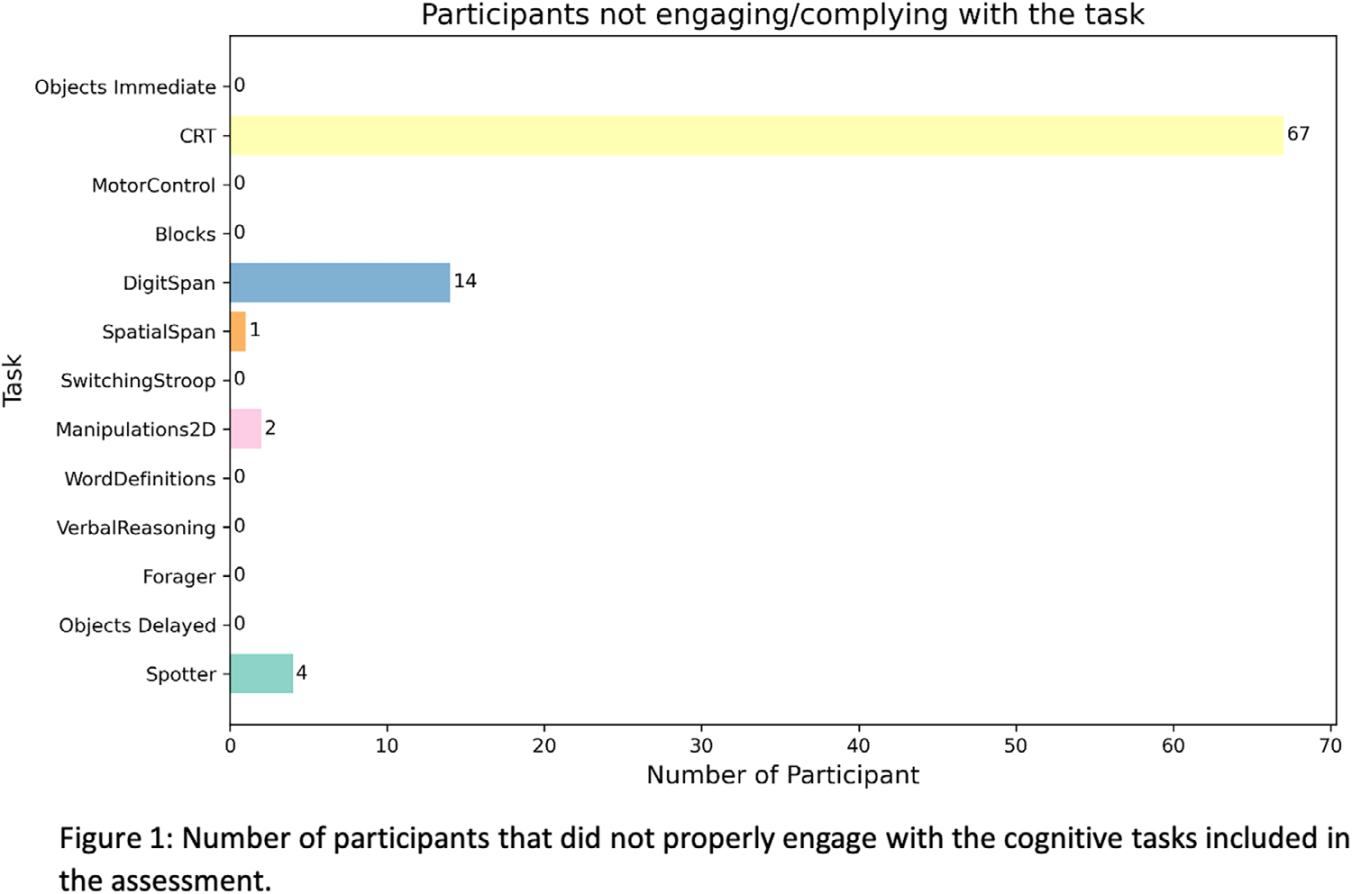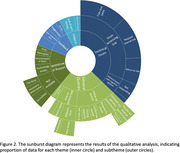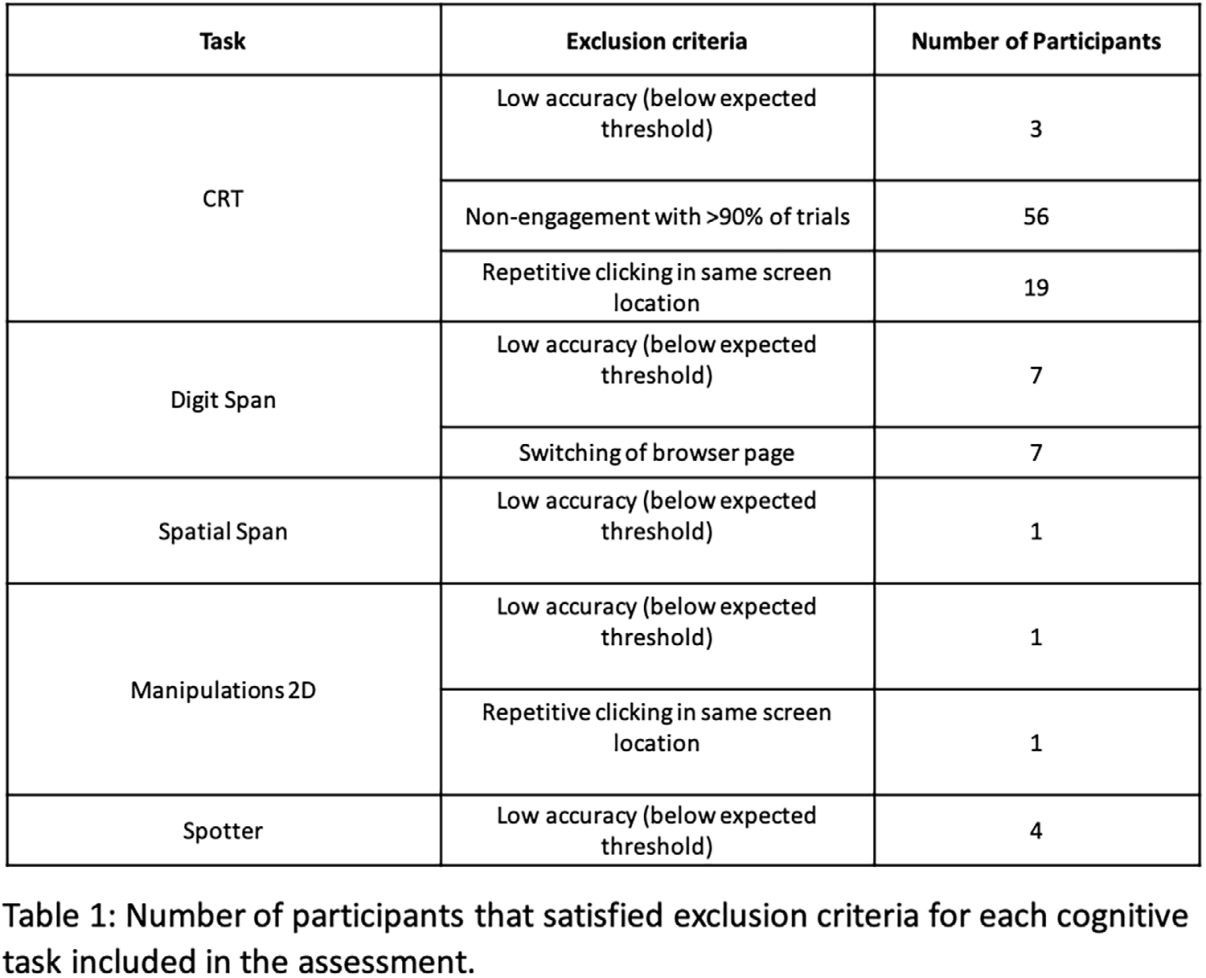# Usability and Compliance of Online Cognitive Assessments in the British 1946 Birth Cohort

**DOI:** 10.1002/alz.091632

**Published:** 2025-01-03

**Authors:** Valentina Giunchiglia, Ziyuan Cai, Martina Del Giovane, Rebecca E Street, Kirsty Lu, Andrew Wong, Maria Popham, Heidi Murray‐Smith, Marcus Richards, Sebastian J. Crutch, Jonathan M. Schott, Adam Hampshire

**Affiliations:** ^1^ Imperial College London, Department of Brain Sciences, London United Kingdom; ^2^ Imperial College London and the University of Surrey, UK Dementia Research Institute Care Research and Technology Centre, London, London United Kingdom; ^3^ Dementia Research Centre, UCL Queen Square Institute of Neurology, University College London, London United Kingdom; ^4^ MRC Unit for Lifelong Health and Ageing at UCL, London United Kingdom; ^5^ MRC Unit for Lifelong Health & Ageing at UCL, London United Kingdom

## Abstract

**Background:**

Cognitive assessments are essential for detecting and monitoring cognitive changes in neurological populations. Compared to standard pen‐and‐paper tests, online cognitive tasks offer a more accessible, scalable, repeatable and cost‐effective approach to assessment. Cognitron is an online cognitive assessment platform with previously demonstrated validity and reliability (1). This study aims to derive meaningful insights into the application of online and computerized assessments in older age cohorts by examine the usability of Cognitron tasks with the MRC National Survey of Health and Development (NSHD; the 1946 British birth cohort).

**Method:**

1753 members of the NSHD cohort (all aged 77) were invited to complete a battery comprising 13 Cognitron tasks, spanning multiple cognitive domains, on their home devices. Each task generated measures of accuracy and reaction time. We examined data quality, completion rates, and levels of engagement and compliance. Qualitative data from email (N = 200) and telephone (N = 78) queries were analysed to determine insights into participant experiences.

**Result:**

990 members (56.47%) consented to participate in the study, of whom 813 attempted the battery (46.34%). 739/813 (90.89%) completed all cognitive tests (average completion time = 40 minutes). 8 tasks (61.53%) were completed by all participants. 4 tasks had <2% of participants incompletely engaging. One task (Choice Reaction Time) had 9% of data with signs of non‐compliance and was dropped from further analysis (Figure 1). The most common signs of non‐compliance were repetitive clicking in the same screen location, switching of browser page, accuracy scores below minimum expected (e.g. failing the simplest trials) and non‐engagement with >90% of trials (Table 1). The qualitative analysis of the reasons participants contacted the study by email or telephone identified five key themes: (1) technical issues (N = 137), (2) general queries (N = 88), (3) reasons for withdrawing (N = 72), (4) providing feedback (N = 43) and (5) subjective reports (N = 28) (Figure 2).

**Conclusion:**

Cognitron assessment was characterised by high completion, engagement and compliance rates in the NSHD cohort, suggesting that online cognitive assessments have potential to monitor cognitive changes in older age cohorts, including those at risk of developing dementia.

**Reference**

(1) Del Giovane, Martina, et al. (2023) *EClinicalMedicine* 59 https://doi.org/10.1016/j.eclinm.2023.101980